# Are we there yet? Exploring astrocyte heterogeneity one cell at a time

**DOI:** 10.1002/glia.24621

**Published:** 2024-09-23

**Authors:** Michael R. O'Dea, Philip Hasel

**Affiliations:** ^1^ Neuroscience Institute NYU Grossman School of Medicine New York New York USA; ^2^ UK Dementia Research Institute at the University of Edinburgh Edinburgh Scotland UK; ^3^ Centre for Discovery Brain Sciences, School of Biomedical Sciences, College of Medicine and Veterinary Medicine The University of Edinburgh Edinburgh Scotland UK

**Keywords:** astrocytes, heterogeneity, single cell RNA‐sequencing, spatial transcriptomics

## Abstract

Astrocytes are a highly abundant cell type in the brain and spinal cord. Like neurons, astrocytes can be molecularly and functionally distinct to fulfill specialized roles. Recent technical advances in sequencing‐based single cell assays have driven an explosion of omics data characterizing astrocytes in the healthy, aged, injured, and diseased central nervous system. In this review, we will discuss recent studies which have furthered our understanding of astrocyte biology and heterogeneity, as well as discuss the limitations and challenges of sequencing‐based single cell and spatial genomics methods and their potential future utility.

## Main Points

- Astrocytes are present throughout the entire central nervous system where they play critical support functions and respond to injury and disease. In both health and disease, astrocytes show strikingheterogeneity.
- 'In this review we discuss how astrocyte heterogeneity is assessed by single cell and spatial transcriptomic approaches, what we have learned about astrocyte subtypes so far and what's still in store in the not‐sodistant future.

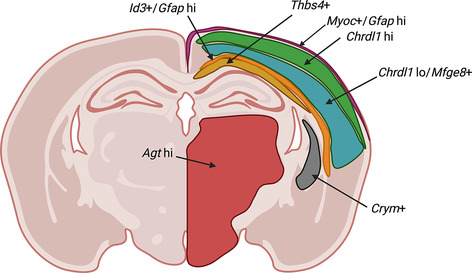

## 1 BACKGROUND OF SEQUENCING MODALITIES AND PLATFORMS TO STUDY ASTROCYTE HETEROGENEITY

That the neuron is a diverse cell type has been evident from the earliest drawings of Ramon y Cajal, which revealed an incredible array of morphologies across different neurons and between different regions of the central nervous system (Ramón y Cajal, S., 1909). In contrast, astrocytes exhibit exceedingly complex branching territories but less striking morphological heterogeneity (Baldwin et al., 2023; Kimelberg, 2009)–giving rise to the impression that these glial cells are relatively homogenous. The advent of single‐cell and spatial transcriptomics has demonstrated conclusively that this assumption of uniformity is incorrect. In different brain regions and across various diseases, astrocytes display a wide spectrum of gene expression profiles which suggest varied functions in health and disease. Profiling this astrocyte diversity has been made possible primarily through the application of next generation sequencing (NGS) technology at the level of the single cell.

NGS sequencing, in which cDNA libraries are fragmented into short reads and sequenced in a massively parallel manner with high fidelity, enabled whole transcriptome sequencing of the first cDNA libraries generated from the RNA of single cells (Tang et al., 2009). Since the generation of these first single cell transcriptomes, both academic labs and commercial entities have developed dozens of different protocols and platforms to generate single cell transcriptomic libraries by partitioning single cells and various reagents into 1000 of discrete reactions. The most frequently used extant methods include 10X Genomics' Chromium platform (Zheng et al., 2017), Drop‐seq (Macosko et al., 2015), InDrop (Klein et al., 2015), Smart‐seq2 (Picelli et al., 2013), and SPLIT‐seq (Rosenberg et al., 2018). These platforms have different features and requirements, allowing researchers to choose between methods of various reagent costs, equipment requirements, protocol complexities, and variable sensitivity and reproducibility. Most of these platforms can be readily applied to both single cells as well as single nuclei, allowing for flexibility in tissue collection and dissociation methods. Innovation in this space shows no sign of slowing, with many of these platforms undergoing regular updates to reagent chemistry or partitioning methods to increase the number of cells that can be profiled in each experiment, and to expand the range of possible input material that can be profiled. For example, adapted versions of the 10X Chromium method along with new methods now allow for assaying gene expression using formaldehyde fixed tissue (Phan et al., 2021; Thomsen et al., 2016). And last year, Fluent Biosciences released a new platform named PIP‐seq using barcoded lipid droplets and vortexing for single cell partitioning which can scale to 1 million single cells per sample (Clark et al., 2023). The rapid up‐scaling of the number of cells which can be profiled at one time using these various assays will be of considerable utility, increasing the power of astrocyte single‐cell profiling experiments to detect meaningful transcriptional differences and improving resolution of rare subpopulations. Further, alterations to existing methods as well as novel approaches now make it possible to apply long read sequencing to single cells (Gupta et al., 2018; Hardwick et al., 2022), with the potential to uncover alternative splicing dynamics and novel transcript isoforms which may be specific to certain cell types or sub‐populations—an axis of variation not well explored in astrocytes to date which could have importance in a number of astrocyte functions. For instance, given the role of ion channel alternative splicing in regulating neuronal plasticity (Li et al., 2020), these techniques may hold promise for uncovering new mechanisms regulating the electrophysiological properties of astrocytes.

While the vast majority of single‐cell genomics platforms and methods are designed to assess the RNA present in single cells at a given point in time (i.e. single‐cell RNA‐seq), recent years have heralded the development of new methods to profiling other genomic features of single cells or nuclei—in particular, identifying regions of accessible chromatin (scATAC‐seq (Buenrostro et al., 2015)), profiling DNA methylation (e.g. snmC‐seq2 (Luo et al., 2018) and scBS‐seq (Smallwood et al., 2014)), assessing chromatin contacts (scHi‐C (Nagano et al., 2013)), examining histone marks and transcription factor binding (scChIP‐seq (Rotem et al., 2015)), and even whole genome sequencing (sc‐WGS (Lan et al., 2017; Vitak et al., 2017; Zahn et al., 2017)). Several “multi‐omic” methods now exist for combinatorial profiling using multiple modalities in the same single cells, with the most common examples marrying scRNA‐seq with scATAC‐seq (such as the 10X Multiome assay, SHARE‐seq (Ma et al., 2020), and SNARE‐seq (Chen et al., 2019)), single cell methylome sequencing (scM&T‐seq (Angermueller et al., 2016), and scMT‐seq (Hu et al., 2019)), proteome profiling (CITE‐seq (Stoeckius et al., 2017)), or single cell DNA sequencing (G&T‐seq (Macaulay et al., 2015), and DR‐seq (Dey et al., 2015)). Multi‐omic single cell technologies continue to develop rapidly, with the newest approaches even allowing for triple modality single cell profiling (scNMT‐seq (Clark et al., 2018) and scTrio‐seq (Hou et al., 2016)). The application of single‐cell epigenetic profiling methods is particularly exciting for the study of astrocyte biology, as these methods can provide a window into the gene regulatory mechanisms underlying astrocyte transcriptional diversity. These methods could reveal novel transcription factor regulators and uncover enhancer or repressor elements which influence homeostatic and reactivity diversity. The multi‐omic methods which allow simultaneous profiling of the transcriptome and epigenome will be crucial for firmly connecting these regulatory mechanisms to gene expression.

Despite the rapidly expanding popularity of these single cell genomics approaches, these methods all have one characteristic flaw: they all require dissociation, and thus destruction of all spatial organization, of the input tissue. While this is not an issue for certain tissue or cell types, for example, peripheral blood mononuclear cells, for studies of the brain this is inherently problematic. Most previous single cell profiling of astrocytes and other brain cells have either used large brain regions or whole brains as input, or microdissection in order to assess whether these cells exhibit regional differences in gene expression or chromatin accessibility. Though these approaches can be useful, they require careful and reproducible dissections across different brain regions and samples to avoid artefactual variation and false discoveries. Additionally, dissociation itself can induce cell stress responses and gene expression artifacts which may mask underlying biological variation (Denisenko et al., 2020; Marsh et al., 2022).

To avoid this loss of spatial information, in situ sequencing‐based spatial transcriptomics were developed to allow for gene expression profiling in intact thin cryosections (Stahl et al., 2016) (the most common method being 10X Genomics' Visium). These methods capture RNAs for library generation using oligonucleotide‐conjugated barcoded spots on a specialized Slides.

After histological staining or immunofluorescence imaging, library preparation can proceed in a similar manner to standard single‐cell sequencing methods, and the resulting sequencing data can be mapped back to its original spatial context due to the spatial barcodes contained in each read pair. These methods have the advantage of allowing unbiased, genome‐wide profiling of gene expression across the tissue section; however, they are characterized by several disadvantages which limit the usefulness of applying these methods to brain tissue (and astrocytes in particular). Namely, each barcoded spot is typically much larger than the size of a cell (55 μm in diameter in the case of Visium), meaning the resulting data is not single‐cell resolution, and these methods suffer from gene dropout resulting in a less comprehensive picture of RNA transcripts present than standard single‐cell transcriptomics methods.

Many computational tools have been developed to enhance the resulting data and minimize these weaknesses (Abdelaal et al., 2020; Biancalani et al., 2021; Elosua‐Bayes et al., 2021; Zhao et al., 2021), with an eye toward inferring gene expression at the level of single cells; and ongoing technological innovation promises to increase the spatial resolution of these methods—such as the newly released Visium HD assay by 10X Genomics, which now features a continuous capture area consisting of 2 × 2 μm barcoded squares (Janesick et al., 2023). Further, these methods have been successfully adapted for spatial profiling of other sequencing‐based genomic modalities, such as ATAC‐seq or ChIP‐seq (e.g. spatial ATAC‐seq and spatial Cut&Tag (Deng, Bartosovic, Kukanja, et al., 2022; Deng, Bartosovic, Ma, et al., 2022; Llorens‐Bobadilla et al., 2023; Zhang, Deng, et al., 2023)).

A plethora of all‐optical alternatives based on in situ hybridization, such as MERFISH (Chen et al., 2015) (commercialized in Vizgen's MERSCOPE system), EEL‐FISH (Borm et al., 2023), 10X Genomics' Xenium (Janesick et al., 2023), Nanostring's CosMx (He et al., 2022), and many others have recently emerged, allowing for gene expression profiling of dozens to 1000 of genes—with the caveat that genes of interest must be chosen in advance. Further, these methods can suffer from errors due to optical crowding of fluorescent signals from adjacent transcripts (Tian, Chen, & Macosko, 2023).

To date, nearly all single‐cell studies examining astrocyte diversity have been conducted with standard single‐cell RNA sequencing using Dropseq or 10X Genomics assays, using either targeted (e.g. FACS or MACS enrichment or de‐enrichment of specific cell types of interest) or untargeted approaches. More recent work has applied sequencing‐based spatial transcriptomics to assess potential astrocyte differences between brain regions and pathological conditions (Hasel et al., 2021) (see below). Currently, only a small number of studies have applied other single‐cell genomics methods and modalities to the study of astrocytes (Burda et al., 2022; Clark et al., 2021; Lee et al., 2024), leaving ample room for expanding genomic studies of astrocyte biology. Here we will review several major single cell and spatial genomics studies and what they can tell us about the biology of astrocytes. We will discuss pitfalls which challenge interpretation of single cell sequencing studies of astrocytes and review outstanding gaps in our understanding which may be filled by future studies. Finally, we will discuss how single cell and spatial genomics approaches and existing data may be leveraged in the future to further our understanding of astrocyte development and function.

## 2 ASTROCYTE HETEROGENEITY THROUGH THE LENS OF TIME

A simple immunohistochemical staining for the astrocytic intermediate filament protein glial fibrillary acidic protein (GFAP) in a healthy mouse brain section can easily show how astrocyte heterogeneity has emerged as its own, now rapidly growing field (Blanco‐Suarez & Allen, 2022; Hasel, Aisenberg, et al., 2023). GFAP has historically been, and to this day still actively is, used as a marker for astrocytes and astrocyte reactivity (Hol & Pekny, 2015). In a healthy mouse brain section, it will readily stain at high intensity white matter tracts, the hippocampus, penetrating blood vessels, and the brain surface just below the pia, but exhibits little signal in most of the cortex or other subcortical regions. Classically, GFAP+ or GFAP‐high astrocytes in the white matter are described as fibrous, while GFAP‐ or GFAP‐low astrocytes in the cortex as protoplasmic, with the latter exhibiting fine leaflets embedded in a bushy and branched morphology. Today, we know that astrocytes can be divided into many more subtypes and substates.

Descriptions of astrocyte heterogeneity, however, date significantly before the use of GFAP in immunohistochemistry. Illustrations from Ramon y Cajal (Ramón y Cajal, S., 1909) and Gustaf Retzius (Retzius, 1881) around the turn of the 19th century have highlighted the vast morphological heterogeneity of astrocytes in higher vertebrates. Illustrations by Gustaf Retzius from the cortex of adult humans show astrocytes with elaborate processes, extending like spider legs from their cell bodies (Retzius, 1881). Some of these astrocytes have processes that extend across several cortical layers, a phenomenon that has been suggested to be specific to hominids (Kwon et al., 2014).

Gustaf Retzius' illustrations already suggested morphological specializations of astrocytes to distinct anatomical domains, including the brain surface and blood vessels where astrocyte cell bodies with unique morphologies attach to these border structures and make up the *glia limitans* (Retzius, 1881). About a century later, morphological specializations to circuits or anatomical domains would be shown to be accompanied by molecular specialization, an observation that was at least in part driven by single cell and spatial transcriptomics approaches. However, despite large scale single cell approaches in the last decade, some histologically defined subtypes that are readily observable in situ still seem to be undefined to this day at the molecular level.

## 3 WHAT DO WE KNOW ABOUT ASTROCYTE HETEROGENEITY IN THE HEALTHY BRAIN TODAY?

Today, astrocytes are thought to adjust their morphology, molecular profile, and function to the circuit or anatomical domain they occupy. Like neurons, astrocytes can show circuit‐ or domain‐specific markers and are hypothesized to specialize to local demands. This can include differential expression of genes involved in neurotransmitter uptake and metabolism such as *Slc6a1*, *Slc6a11*, *Slc1a2, Slc1a3*, *Aldh5a1*, and *Glul* depending on the preferential release of glutamate or GABA within specific circuits (Boisvert et al., 2018; Chai et al., 2017; Hasel et al., 2021), expression of synapto‐regulatory proteins, such as *Chrdl1* across different cortical layers and developmental time points to shape neuronal development (Bayraktar et al., 2020; Blanco‐Suarez et al., 2018) or subtypes or substates of astrocytes restricted to local pathologies, such as plaque depositions in Alzheimer's disease (Castranio et al., n.d.; Chen et al., 2020; Habib et al., 2020). This specialization is at least in part driven by neuronal inputs, since astrocytes implanted into mouse brains acquire the locally appropriate morphologies (Preman et al., 2021), and neurons and their activity states are known to alter astrocytic identities *in vitro* (Hasel et al., 2017) and *in vivo* (Farmer et al., 2016).

Initial scRNA‐seq studies capturing astrocyte heterogeneity described two cortical subtypes of astrocytes that broadly fell into subtypes either expressing high or low levels of the astrocyte marker genes *Gfap* and *Aqp4*, with the high expressors occupying the upper layers of the mouse cortex (Zeisel et al., 2015). The same group later showed additional subtypes of astrocytes across the mouse brain, highlighting clear demarcations between telencephalic and diencephalic brain regions, which could be broadly distinguished by the astrocytic expression of either *Mfge8* or *Agt*, respectively (Zeisel et al., 2018).

A set of landmark articles in 2019 and 2020 (Batiuk et al., 2020; Bayraktar et al., 2020; Hodge et al., 2019) used a combination of single cell or nucleus RNA‐seq and spatially resolved gene expression profiling to establish that astrocytes show differential gene expression profiles across the cortical layers in both mouse and human. Within the cortical layers L1‐L6, astrocyte heterogeneity appeared to exist on a transcriptomic gradient, with clear cut offs at L6/white matter and L1 boundaries (Bayraktar et al., 2020; Hodge et al., 2019). Interestingly, Layer I astrocytes exist in a more similar transcriptomic space to white matter astrocytes than to other physically much closer gray matter astrocytes, with high expression of genes such as *Id3* and *Gfap* (Bayraktar et al., 2020; Hodge et al., 2019). Recently, the discovery of surface or *glia limitans superficialis* (GLS) astrocytes showed further that while distinct from each other, white matter and GLS astrocytes exhibit many transcriptomic similarities (Hasel et al., 2023). Critically, astrocytes in L1 are distinct from GLS astrocytes in many aspects, including striking differences in morphology and gene expression (Batiuk et al., 2020; Bayraktar et al., 2020; Hasel et al., 2023).

**FIGURE 1:**
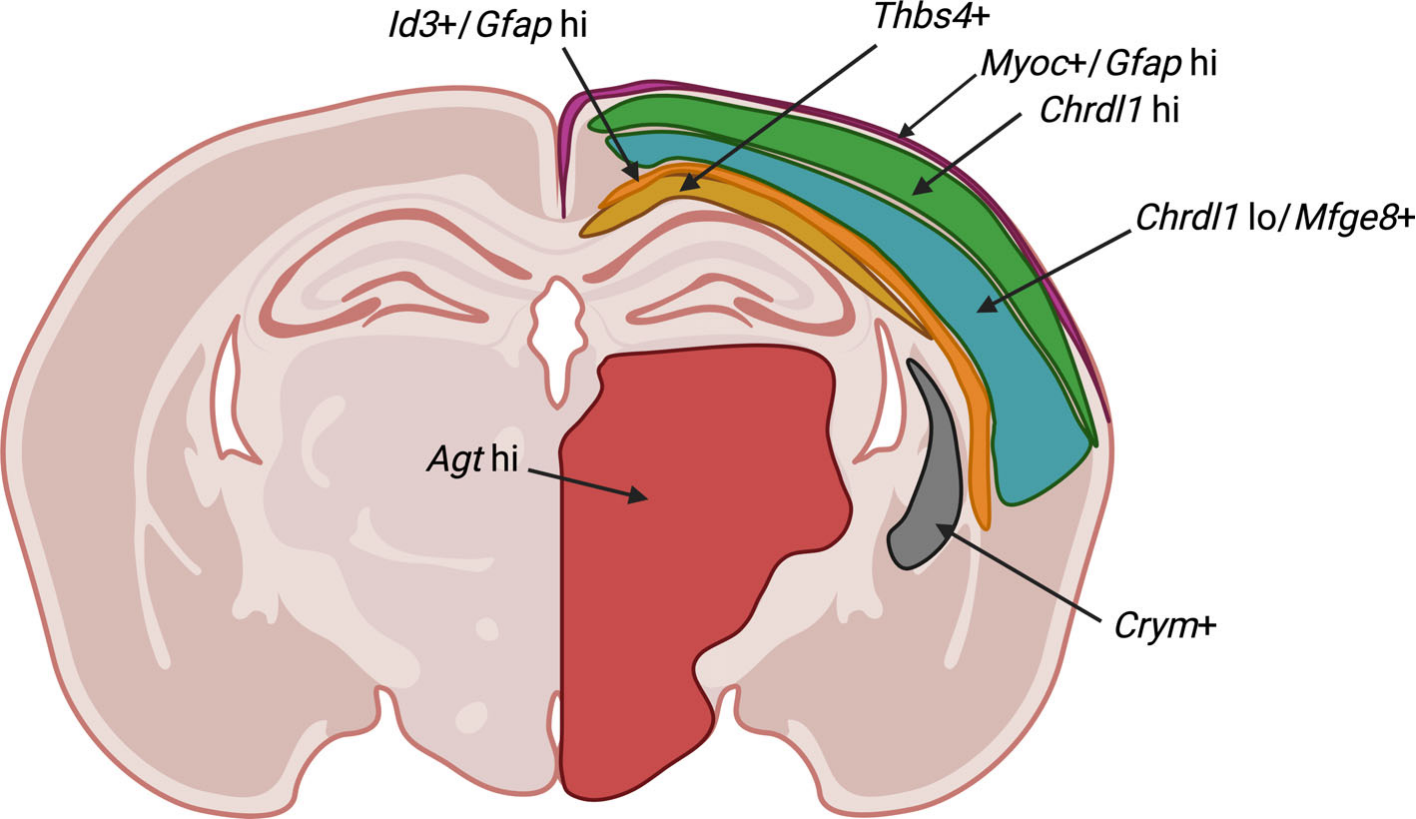
*Single cell*/*nucleus RNA*‐*seq experiments combined with spatial transcriptomics have uncovered discrete, locally restricted subtypes of astrocytes*. *While consensus is still incomplete*, this list includes *Crym* + striatal astrocytes (Chai et al., 2017), diencephalic *Agt* + astrocytes (Zeisel et al., 2018), *Chrdl1* hi astrocytes in L2‐L4, *Id3*+/*Gfap* + astrocytes in white matter and L1 (Bayraktar et al., 2020), *Thbs4*+ white matter, and periventricular astrocytes (Ardaya et al., 2023; Hasel et al., 2023), as well as *Myoc* + *glia limitans superficialis* astrocytes (Hasel et al., 2023).

An elegant study from Endo and colleagues (Endo et al., 2022) recently combined deep bulk RNA‐seq with region‐specific scRNA‐seq and morphological studies in an effort to link molecular profiles to astrocyte morphology and local circuit functions, expanding on the concept that astrocytes show circuit‐ or domain‐specific molecular specialization. This approach uncovered modules of astrocyte subtype‐ and region‐specific gene expression which correlated with various morphological parameters, suggesting molecular mechanisms underlying the diverse astrocyte morphologies present across different anatomical domains. The same group previously reported that astrocytes within the striatum selectively express *Crym* (Chai et al., 2017), and later showed that Crym in striatal astrocytes affects mouse behaviour (Ollivier et al., 2024). Another approach to link astrocyte morphology to single‐cell transcriptomes using a modified version of Patch‐seq in human fetal tissue was recently described (Allen et al., 2022), which holds promise for application to a wider array of brain regions and ages (Figure 1).

Other than cortical, striatal and surface subtypes, white matter tracts are occupied by molecularly distinct astrocytes subtypes. This includes astrocytes expressing high levels of *Gfap* and *Id3* (Bayraktar et al., 2020; Hasel et al., 2021; Hodge et al., 2019) and/or *Thbs4* (Ardaya et al., 2023; Hasel et al., 2023). The latter subtype also occupies periventricular domains and were recently shown to be capable of migration to even distal scar border regions after stroke (Ardaya et al., 2023). Subtypes of subventricular astrocytes (or astrocyte‐like cells) have also been found by the Doetsch lab using scRNA‐seq and validated in situ, suggesting that the regional distribution of astrocyte subtypes around the subventricular zone defines their cell type fate (Mizrak et al., 2019).

As the list of astrocyte molecular subtypes continues to grow, the question of how these molecular differences emerge looms ever larger. Recent studies indicate a role for both developmental lineage and local environment cues in imposing astrocyte molecular phenotypes. Fate mapping in mice and human primary tissue demonstrates that astrocytes arising from different progenitor pools seed different areas of the neocortex and may maintain morphological and transcriptional differences in adulthood (Allen et al., 2022; Zhou et al., 2023). In an example of microenvironmental cues driving astrocyte molecular identity, a recent study using single‐nucleus RNA‐seq, MERFISH, and cell transplantations implicated local expression of Sonic hedgehog (Shh) in the development of septum sub‐region astrocyte sub‐populations (Xie et al., 2023). While these studies have evaluated a select few progenitor pools and secreted cues, this is surely the tip of the iceberg of mechanisms influencing astrocyte molecular diversity.

While astrocytes show this heterogeneity discussed above, some astrocytic genes can still be used as brain‐wide canonical cell type markers nonetheless. Both in mouse (Zeisel et al., 2018) and human (Hodge et al., 2019) *Aqp4* is pan‐astrocytic and can be used as a cell type marker, as well as *Slc1a3* and *Aldh1l1* (Bayraktar et al., 2020; Hasel et al., 2021).

Recently, large‐scale single‐cell/nucleus and spatial atlases released under the Brain Research through Advancing Innovative Neurotechnologies (BRAIN) Initiative—Cell Census Network (BICCN) have significantly extended our knowledge of astrocyte heterogeneity at both the cellular and spatial level in mouse and human. For mouse astrocytes, this includes large scale sc/snRNA‐seq combined with spatial transcriptomics using MERFISH, STARmap PLUS or Slide‐seq, which include genes demarcating several astrocyte subtypes (Langlieb et al., 2023; Shi et al., 2023; Yao et al., 2023; Zhang, Pan, et al., 2023), as well as single‐cell methylome, multiome, and/or chromatin conformation (Liu et al., 2023; Zemke et al., 2023; Zu et al., 2023). For human astrocytes, this includes sc/snRNA‐seq of the developing pre‐natal hypothalamus (Herb et al., 2023) and post‐natal cerebellum (Ament et al., 2023), sn/scRNA‐seq or multiome sequencing of the pre‐ and post‐natal cortex (Jorstad, Close, et al., 2023; Velmeshev et al., 2023; Zhu et al., 2023), adult middle temporal gyrus (Jorstad, Song, et al., 2023), single cell 3D chromatin structure and DNA methylation from 46 brain regions (Tian, Zhou, et al., 2023), snATAC‐seq on multiple brain regions (Li et al., 2023) as well as MERFISH and snRNA‐seq of the developing pre‐natal thalamus (Kim, Shin, et al., 2023).

In particular, Siletti et al (Siletti et al., 2023). have generated snRNA‐seq data from 10 brain regions of the human brain, sequencing more than 150,000 astrocytes. In the cortex, they suggested they could partition astrocytes broadly into white matter (TNC+), gray matter (WIF1+), and interlaminar astrocytes (LMO2+), which validation in intact tissue will need to confirm in future studies.

While the majority of studies and marker genes mentioned in this section have been focused on adult tissues, it is important to consider animal age and developmental stage when examining astrocyte heterogeneity. Astrogenesis occurs in the late stages of embryonic development, and in the mouse, astrocytes are thought to mature into adult morphologies and mature molecular states in the postnatal period between birth and approximately 1 month of age (Akdemir et al., 2020; Farhy‐Tselnicker et al., 2021). It is not yet clear precisely when in development many of the above‐described regional gene expression signatures are induced, and additional studies will be required to answer this question—and to evaluate whether these populations persist throughout aging and disease.

Care should be taken when associating differentially expressed genes from sc/snRNA‐seq experiments with functionally distinct or regionally‐restricted astrocyte subtypes. Rigorous validation in situ or in vivo is necessary to not confound the expression of a gene within an astrocyte as assessed by sc/snRNA‐seq experiments with a mal‐defined function or anatomical domain. Descriptions of markers for vessel‐associated astrocyte subtypes, midbrain‐specific astrocytes or hominid‐specific interlaminar astrocytes, for example, are exciting, but encourage validation of these subtype identities for a research community that so heavily relies on bona fide subtype markers to study astrocyte biology.

## 4 WHAT DO WE KNOW ABOUT ASTROCYTE HETEROGENEITY IN INJURY AND DISEASE?

In disease, astrocytes undergo what is called a reactive transformation, often referred to as astrocyte reactivity or (reactive) astrogliosis. Astrocytes will respond to a pathological insult by changing their molecular make‐up, morphology, and function. Depending on the type of insult, this can entail a loss of homeostatic support functions or the gain of functions, including a gain of neurotoxicity (Guttenplan et al., 2020). Historically, these transformations have been described as binary and uniform, where astrocytes either exist in a resting or reactive state, a distinction often made solely by the expression levels of GFAP. However, there is now overwhelming evidence based on single‐cell and spatial transcriptomic studies that astrocytes not only show vast heterogeneity at rest but can fall into dynamic states of reactivity following an insult. It is not entirely clear whether it is the subtype identity at rest, the type of insult, or the anatomical domain that decides the type of reactive transformation—or whether it is a combination of all these factors.

What is apparent is that the locus and history of the insult or injury can create localized reactive transformations. For example, single‐cell and spatial transcriptomics approaches have shown that mice undergoing LPS‐induced inflammation exhibit peri‐vascular reactive astrocyte subtypes induced by interferons (Hasel et al., 2021), and stab wound injury induces similar peri‐injury restricted transformations (Zamboni et al., 2020). In mouse models of Alzheimer's disease, plaque depositions are associated with local reactive transformations (Castranio et al., n.d.; Chen et al., 2020; Grubman et al., 2019; Habib et al., 2020), and both stroke and spinal cord injury can create specific subtypes depending on the distance from the injury site (Kim, Marchildon, et al., 2023; Sarah et al., 2024). Single‐cell and spatial transcriptomics have highlighted in animal models of multiple sclerosis that astrocytes show distinct reactive transformations (Wheeler et al., 2020) that depend on their distance and the activity history of lesion sites (Jing‐Ping et al., 2023; Langseth et al., 2023).

While these localized transformations are most apparent in both single‐cell and spatial transcriptomics studies, astrocytes can also show signs of a “general reactivity” that are not obviously linked to a focal injury. Mice injected with LPS, for example, undergo brain‐wide reactive transformations as shown by the near pan‐astrocytic increased expression of genes such as *Lcn2* and *Serpina3n*, while at the same time showing white‐matter restricted induction of *Timp1* and the induction of *Igtp* in a subset of layer 1 astrocytes (Hasel et al., 2021). These white matter specific transformations are also observed in the aging human brain as has been recently assessed by combining snRNA‐seq and MERFISH (Allen et al., 2023). Critically, absence of specific markers of reactivity cannot necessarily be interpreted as complete absence of reactivity. For example, mild traumatic brain injury can cause the loss of expression of homeostatic proteins in astrocytes without upregulating expression of GFAP (Shandra et al., 2019).

Reactive transformations of astrocytes are often described as homeostatic subtypes taking on a reactive phenotype, but retaining their baseline gene expression profile, or as completely new subtypes whose homeostatic identity is lost due to their severe transformation. For example, peripherally‐induced brain inflammation will cause a reduction in astrocyte subtype marker genes, such as the marker for *glia limitans superficialis* astrocytes *Myoc*, without overwriting their core homeostatic identity, while simultaneously creating new reactive subtypes whose homeostatic identity appears completely lost (Hasel et al., 2021; Hasel et al., 2023). The latter might be a consequence of generally low sequencing depth/gene dropout in sc/snRNA‐seq experiments, and orthogonal methods or additional modalities are needed to understand how subtypes of astrocyte became reactive throughout a disease progression, given that reactive transformations can be highly dynamic across the trajectory of a disease or injury (Habib et al., 2020; Hasel et al., 2021; Kim, Marchildon, et al., 2023).

This latter aspect, the trajectory of a reactivity‐inducing insult across time, is crucial to consider when conducting single‐cell and spatial genomics studies, as the molecular changes associated with astrocyte reactivity can vary dramatically depending on the time point chosen post‐insult. This is particularly evident in focal injuries such as stroke, in which reactivity is rapidly induced, and astrocyte gene expression changes dramatically in the days following the insult. For example, Kim et al (Kim, Marchildon, et al., 2023) described a small subset of astrocytes with a proliferative gene signature present specifically at 3 days post‐photothrombotic stroke, and this population was not observable at earlier or later time points. This highly dynamic temporal dependence of astrocyte reactivity is also readily observable in the LPS model of acute inflammation, in which astrocyte exhibit altered gene expression for several days following the inflammatory insult—yet the particular genes which are differentially expressed vary greatly at different time points (Diaz‐Castro et al., 2021; Hasel et al., 2021). What the underlying mechanisms are which drive these temporal changes is an open question. These temporal differences may represent different phases of astrocyte reactivity (or recovery to a homeostatic state) and may impart distinct functions, making this area a promising avenue for therapeutic investigation.

Indeed, while these sequencing technologies are powerful discovery tools, particularly when adding spatial genomics approaches, the data generated should be interpreted carefully and validated thoroughly. There is significant therapeutic potential in targeting reactive subtypes of astrocytes in disease, but it ultimately relies on functional characterization of subtypes using genetic approaches. This can include reporter mice for astrocyte subtypes occupying strategic locations in the brain, such as perivascular astrocytes (Morales et al., 2022), mice conditionally expressing fluorescent reporter proteins in subsets of reactive astrocytes, like possible with the *Lcn2*‐CreERT2 mouse (Agnew‐Svoboda et al., 2022), or mice lacking the ability to transform into specific reactive subtypes (Liddelow et al., 2017).

## 5 THE CHALLENGES AND PITFALLS OF STUDYING ASTROCYTE HETEROGENEITY USING SEQUENCING TECHNOLOGIES

While single cell genomics assays have been incredibly powerful tools for profiling the molecular heterogeneity of astrocytes and other glial cells, the application of these methods and the analysis of resulting data present major challenges which can limit the utility of these methods.

One of the primary challenges of applying single cell sequencing to study astrocyte biology is the inconvenient fact that astrocytes are typically recovered from single cell dissociations at lower rates than their prevalence in the brain in situ. While some reports suggest capture of astrocytes and other cell types may improve with single nucleus dissociation methods compared to whole cell isolations (Oh et al., 2022; Santiago et al., 2023), these poor capture rates suggest there are some inherent properties of these cells which make them difficult to isolate and partition for single cell assays. This may be a result of the many fine processes which astrocytes extend into the surrounding neuropil. Whatever the cause, the practical result of this feature is that untargeted single cell assays (those which use all cells in the input tissue) often exhibit low astrocyte numbers and reduced power to detect rare subpopulations or disease‐specific substates. Alternatively, targeted assays can be used to enrich specifically for astrocytes, most commonly by fluorescence activated cell or nuclei sorting (FACS or FANS) using astrocyte‐specific fluorescent reporter mouse lines, such as the Aldh1l1‐EGFP BAC transgenic strain or the Aldh1l1‐bacTRAP strain from Nathaniel Heintz's lab (Doyle et al., 2008; Gong et al., 2003; Hasel et al., 2021; Kim, Marchildon, et al., 2023), or using immunofluorescence staining for astrocyte cell surface antigens like GLAST/ACSA‐1 and ATP1B2/ACSA‐2 or nuclear markers like SOX9+ (Batiuk et al., 2020; Burda et al., 2022; Sadick et al., 2022). Alternatively, magnetic activated cell sorting (MACS) targeting similar surface antigens can also be used to enrich for astrocytes when sorting whole cells (Batiuk et al., 2017). These methods can greatly increase astrocyte capture and thus power to detect differential gene expression between subpopulations or disease states, though at the expense of increased time and cost.

Caution is also warranted when working with live cells, which may exhibit transcriptional perturbations induced by dissociation, FACS, or MACS (Denisenko et al., 2020; Marsh et al., 2022; van den Brink et al., 2017). This ex vivo alteration of gene expression is most strongly induced in microglia, but may also occur in astrocytes as well (Marsh et al., 2022). Use of transcription and translation inhibitors, methanol or formaldehyde fixation, or instead using a single‐nucleus approach are all potential options for limiting dissociation‐ and sorting‐related cellular changes (van den Brink et al., 2017)—however these approaches could also alter cellular viability, integrity, or transcription. At a minimum, researchers applying single‐cell RNA‐seq ought to compare their single astrocyte transcriptomes to previously defined ex vivo activation gene signatures (e.g. in Marsh et al (Marsh et al., 2022).) to assess the possibility of artefactual gene expression changes.

Recent large‐scale mouse brain single cell RNA sequencing, single nucleus ATAC sequencing, and MERFISH spatial transcriptomics atlases generated by the BRAIN Initiative Cell Census Network (BICCN) hold great promise for examining astrocyte heterogeneity between and within brain regions, and indeed these studies identified notable gene expression and chromatin accessibility differences between astrocytes in different brain regions. Yet some caution must be taken in the interpretation of atlases of this incredible scale. First, while these atlases contain 100 of 1000 of single astrocyte transcriptomes and epigenomes, the number of astrocytes profiled per sample is low compared to previous datasets. This is likely attributable to most samples in these atlases undergoing FACS or FANS to enrich for neurons, resulting in depletion of astrocytes. In addition to this decreased astrocyte cell capture, a recent study demonstrated that untargeted and neuron‐enriching single cell datasets typically contain significant neuron‐derived ambient RNA, which contaminates the gene expression signatures of less abundant glial cell populations and confounds interpretation (Caglayan et al., 2022). Computational approaches which estimate and correct for ambient RNA contamination may help mitigate these issues (Fleming et al., 2023; Young & Behjati, 2020). Further, large MERFISH spatial transcriptomics datasets included in these atlases can be used to evaluate expression of candidate genes in astrocytes in intact tissue, yet the problem of mis‐attributed neuronal gene expression is not completely resolved with this approach due to the challenge of accurate cell segmentation (David et al., 2023; Hartman & Satija, 2024). Because of these challenges, we still view astrocyte enrichment by FACS, FANS, MACS, or other methods as crucial for high quality single cell genomics studies of astrocyte diversity and function.

Perhaps the biggest challenge for the use of single cell genomics in examining astrocyte heterogeneity is the subjectivity of identifying astrocyte subtypes and substates in the resulting data. Almost all existing datasets utilize a common set of user‐friendly single cell analysis tools such as scanpy (Wolf et al., 2018) or Seurat (Hao et al., 2024) (or scATAC‐seq tools such as ArchR (Granja et al., 2021), SnapATAC (Fang et al., 2021), and Signac (Stuart et al., 2021)) which, though powerful, typically apply k‐means clustering methods in which the resulting number of clusters is determined by a user‐supplied resolution parameter. The choice of this resolution parameter, despite being central to the identification of purported astrocyte subpopulations or disease‐associated sub‐states, is often applied with an arbitrary default or a subjective user‐chosen value (Kiselev et al., 2019). Further, these analysis tools allow the user to endlessly sub‐cluster cells into ever smaller sub‐groups even in the absence of underlying structure (Scott et al., 2023). While user input, informed by the expertise of the investigator, can be useful for identifying potentially meaningful astrocyte diversity, this also introduces bias into the analysis. Several computational approaches have emerged for the automated or semi‐automated clustering of single‐cell data (Kiselev et al., 2017; Petersen et al., 2024; Scott et al., 2023), and application of these tools to astrocyte single‐cell genomics data may be helpful for generating more robust and objective clustering results.

In any case, what represents a “real” or “meaningful” astrocyte subpopulation or state in single‐cell sequencing data may be an inherently subjective question. Though reviewers often demand evidence of related protein expression as validation of the “realness” of a purported astrocyte cluster, this is not always possible due to technical reasons, such as a lack of suitable antibodies, or discordance between transcription and translation kinetics. Additionally, given the wealth of studies demonstrating RNA functions in the absence of downstream protein translation, such as regulation of transcription, splicing, and translation (Mattick & Makunin, 2006), it is worth considering whether gene expression differences alone might underlie functional differences among some astrocytes populations. We envision that adequately assessing the molecular diversity of astrocytes from single‐cell genomics data in future studies will require multi‐faceted validation with orthogonal approaches. This may entail inclusion of additional sequencing modalities (e.g. multi‐omic single cell methods), paired sequencing‐based or optical spatial transcriptomics, in situ hybridization, or immunohistochemistry, as well as genetic and pharmacological perturbation experiments.

## 6 ARE WE THERE YET? WHAT IS IN STORE FOR ASTROCYTE HETEROGENEITY IN THE FUTURE

Single‐cell genomics experiments have often received criticism for solely discovering “heterogeneity,” without necessarily adding new insights into the biology or function of astrocytes or other cell types. Given the high price points for these experiments, one could get the impression that single‐cell approaches are simply throwing money at this brain glue in the hopes that something sticks. Indeed, the value of these experiments is not always immediately apparent if single cell approaches are the sole experiment or the primary endpoint of a study. Yet, when carefully applied and cautiously interpreted, single‐cell RNA sequencing and other single‐cell genomics methods give the opportunity to view a snapshot of an astrocyte's biology across 1000 of dimensions (genes) in a single experiment—an undeniably useful opportunity which could prove informative for understanding the role of various astrocyte subtypes and molecular states in brain function and dysfunction. Maximizing the utility of these experiments, with the ever‐growing number of astrocytes sequenced across species, modalities, diseases and brain regions, will require thorough validation efforts and rigorous functional interrogation experiments. These challenging aims will be aided by efforts to increase the accessibility of genomics data and greater collaboration between single‐cell data generators and other astrocyte biologists. Computational integration of the many individual datasets of this rapidly expanding astrocyte single‐cell corpus also has the potential to promote detection of biologically relevant but potentially rare subtypes or reactive substates across smaller datasets, and thus can be used as powerful discovery tools in astrocyte biology—but findings again need to be validated in intact tissue.

Some astrocyte subtypes described in previous studies seem to still be missing from single‐cell genomic atlases, including perivascular astrocytes. Part of the reason for the lack of coverage may be the fact that these subtypes may occupy especially regionally restricted domains and are therefore extremely rare relative to the broader astrocyte population. This, in combination with generally poor capture rates of astrocytes in unsorted single‐cell genomic experiments and the relatively few sequencing studies which have included non‐telencephalic brain regions, leaves our journey to a complete astrocyte single‐cell atlas yet to be completed.

While we are getting closer to a pan‐astrocytic cell genomic library, particularly in mouse, the heterogenous transformations of astrocytes in disease remain understudied. Indeed, homeostatic astrocyte subtypes can show subtype‐specific transformations that depend both on their anatomical position and, critically, the type and duration of insult and can result in entirely new subtypes of reactive astrocytes. The astrocyte responses of great many disease models have yet to be profiled by single‐cell genomics, and uncovering the full spectrum of molecular responses across these diverse pathologies will require many additional single‐cell genomics studies. These studies will benefit immensely from comparisons with prior atlases of homeostatic astrocyte diversity. The identification of disease or subtype‐specific reactive transformation from these types of studies can and has led to the development of genetic tools to understand their molecular make up and function – and will ultimately uncover their contributions to disease progression. There is significant potential in therapeutically exploiting reactive astrocyte subtypes in disease and their bona fide identification is a critical, and ongoing, first step.

Perhaps the most consequential lesson we have learned from the large corpus of sc/snRNA‐seq and spatial transcriptomics experiments profiling astrocytes over the past several years is that astrocyte transcriptional heterogeneity comes hand in hand with each astrocyte's position in the brain. This diversity correlates with discrete brain regions, circuits, and other specialized anatomical domains, such as the brain surface and ventricles. While the underpinnings of this molecular specialization remain to be discovered, this diversity is sure to be imbued at least in part by local neuronal and non‐neuronal inputs and demands, allowing astrocytes to perform specialized local functions. How this inherent heterogeneity relates to the vast range of astrocyte transformations observed in disease is not entirely clear, but we postulate this likely derives from a combination of the type of insult/disease, the location of the pathology, and the competence of each specialized astrocyte subtype to respond to the triggering stimulus. Exploring these mechanisms and interactions will be an active and exciting area of astrocyte biology in the coming years.

## AUTHOR CONTRIBUTIONS

Both P.H. and M.R.O. conceived and wrote the manuscript.

## FUNDING INFORMATION

P.H. is funded through a Wellcome Trust Career Development Award [227287/Z/23/Z]. This work is supported by the UK Dementia Research Institute [award number UK DRI‐CF2023\5] through UK DRI Ltd, principally funded by the UK Medical Research Council.

## Data Availability

No new data generated in this review.
